# Hexa­kis­(1-benzyl-1*H*-imidazole-κ*N* ^3^)manganese(II) bis­(perchlorate)

**DOI:** 10.1107/S1600536811023531

**Published:** 2011-06-22

**Authors:** Gui-Ying Dong, Tong-Fei Liu, Cui-Hong He, Xiao-Chen Deng, Xiao-Ge Shi

**Affiliations:** a College of Chemical Engineering, Hebei United University, Tangshan 063009, People’s Republic of China; b Qian’an College, Hebei United University, Tangshan 063009, People’s Republic of China

## Abstract

In the title compound, [Mn(C_10_H_10_N_2_)_6_](ClO_4_)_2_, the Mn^II^ ion, located on an inversion center, is coordinated by six N atoms from three pairs of symmetry-related 1-benzyl-1*H*-imidazole ligands in a distorted octa­hedral geometry. In the crystal, weak inter­molecular C—H⋯O hydrogen bonds link the complex cations and perchlorate anions.

## Related literature

For background to the coordination chemistry of imidazole and its derivatives, see: Cui *et al.* (2005 ▶); Fan *et al.* (2005 ▶); Li *et al.* (2009 ▶); Peng *et al.* (2010 ▶); Santoro *et al.* (2000 ▶). For the synthesis of 1-benzyl-1*H*-imidazole, see: Shen *et al.* (2010 ▶).
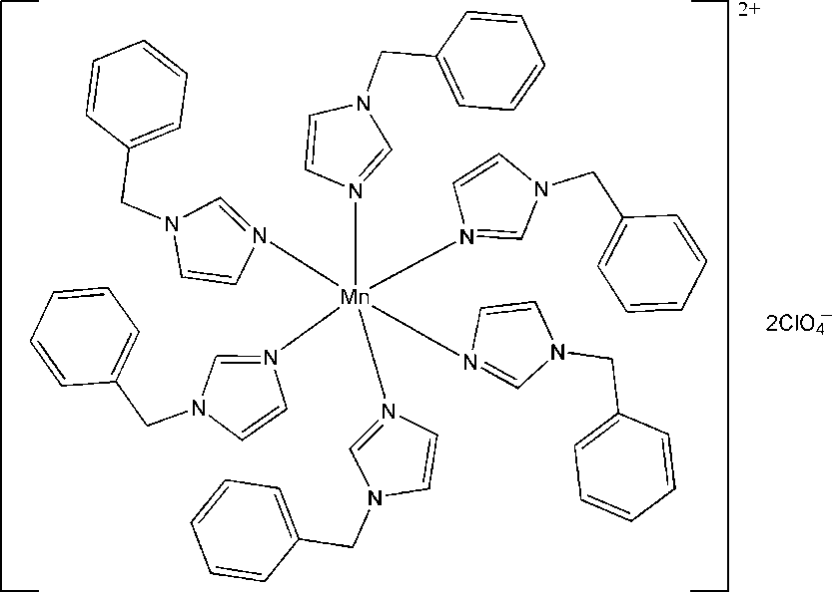


## Experimental

### 

#### Crystal data

[Mn(C_10_H_10_N_2_)_6_](ClO_4_)_2_
*M* *_r_* = 1203.04
Triclinic, 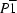

*a* = 9.2832 (19) Å
*b* = 12.744 (3) Å
*c* = 13.317 (3) Å
α = 84.55 (3)°
β = 79.56 (3)°
γ = 75.87 (3)°
*V* = 1500.4 (6) Å^3^
*Z* = 1
Mo *K*α radiation
μ = 0.37 mm^−1^
*T* = 295 K
0.28 × 0.27 × 0.26 mm

#### Data collection

Bruker APEX CCD diffractometer
Absorption correction: multi-scan (*SADABS*; Sheldrick, 1996 ▶) *T*_min_ = 0.796, *T*_max_ = 0.808
15862 measured reflections
6832 independent reflections
5066 reflections with *I* > 2σ(*I*)
*R*_int_ = 0.039

#### Refinement

*R*[*F* ^2^ > 2σ(*F* ^2^)] = 0.062
*wR*(*F* ^2^) = 0.188
*S* = 0.84
6832 reflections
376 parameters
H-atom parameters constrained
Δρ_max_ = 0.34 e Å^−3^
Δρ_min_ = −0.62 e Å^−3^

### 

Data collection: *SMART* (Bruker, 2007 ▶); cell refinement: *SAINT* (Bruker, 2007 ▶); data reduction: *SAINT*; program(s) used to solve structure: *SHELXS97* (Sheldrick, 2008 ▶); program(s) used to refine structure: *SHELXL97* (Sheldrick, 2008 ▶); molecular graphics: *SHELXTL* (Sheldrick, 2008 ▶); software used to prepare material for publication: *SHELXTL*.

## Supplementary Material

Crystal structure: contains datablock(s) I, global. DOI: 10.1107/S1600536811023531/hy2441sup1.cif

Structure factors: contains datablock(s) I. DOI: 10.1107/S1600536811023531/hy2441Isup2.hkl

Additional supplementary materials:  crystallographic information; 3D view; checkCIF report

## Figures and Tables

**Table 1:** Selected bond lengths (Å)

Mn1—N1	2.158 (2)
Mn1—N3	2.158 (2)
Mn1—N5	2.181 (2)

**Table 2:** Hydrogen-bond geometry (Å, °)

*D*—H⋯*A*	*D*—H	H⋯*A*	*D*⋯*A*	*D*—H⋯*A*
C1—H1*A*⋯O1	0.93	2.49	3.286 (4)	144
C14—H14*A*⋯O4	0.97	2.53	3.461 (6)	160
C21—H21*A*⋯O3	0.93	2.56	3.371 (5)	145
C24—H24*B*⋯O1^i^	0.97	2.53	3.469 (5)	164

Symmetry code: (i) 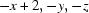.

**Fig. 1.**
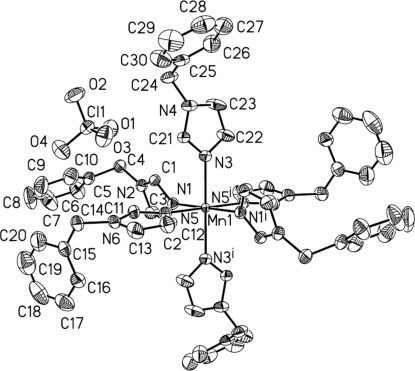
The molecular structure of the title compound, showing displacement ellipsoids at the 30% probability level. H atoms have been omitted for clarity. [Symmetry code: (i) -x+2, -y+1, -z].

**Fig. 2.**
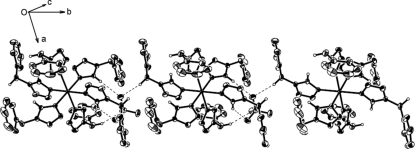
A packing diagram of the title compound. Hydrogen bonds are shown as dashed lines. H atoms not involved in hydrogen bonds have been omitted.

## supplementary crystallographic information

### Comment

Over the past few years, great attention has been paid to the coordination chemistry of imidazole and its derivatives because these compounds are ubiquitous in biological and biochemical structures and functions, such as the roles of histidine as a metal ion binding site in metalloenzymes and in the catalytic mechanism of ribonucleases and other phosphoesterases (Cui *et al.*, 2005; Fan *et al.*, 2005; Li *et al.*, 2009; Peng *et al.*, 2010; Santoro *et al.*, 2000). We report here the crystal structure of the title compound.

The coordination geometry around the Mn^II^ atom is slightly distorted octahedral, defined by six N atoms from six 1-benzyl-1*H*-imidazole ligands (Fig. 1). The Mn—N bond distances lie in a range from 2.158 (2) to 2.181 (2) Å (Table 1). In the crystal, the complex cations and perchlorate anions are linked *via* weak C—H···O hydrogen bonds (Table 2, Fig. 2), which stabilize the structure.

### Experimental

A mixture of MnCl_2_.6H_2_O (197 mg, 1 mmol), salicylic acid (138 mg, 1 mmol), NaOH (40 mg, 1 mmol) and 1-benzyl-1*H*-imidazole (158 mg, 1 mmol) (Shen *et al.*, 2010) in H_2_O (15 ml) was placed in a Teflon-lined stainless vessel and heated to 413 K for 72 h. Then, the reaction system was cooled to room temperature during 24 h to give rise to yellow crystals, which were collected and washed with water (yield: 0.040 g, 20%). Analysis, calculated for C_60_H_60_Cl_2_MnN_12_O_8_: C 59.90, H 5.03, N 13.97%; found: C 59.75, H 4.95, N 13.78%.

### Refinement

H atoms were placed in calculated positions and refined as riding atoms, with C—H = 0.93 (CH) and 0.97 (CH_2_) Å and with *U*_iso_(H) = 1.2*U*_eq_(C).

### Crystal data

**Table:**

[Mn(C_10_H_10_N_2_)_6_](ClO_4_)_2_	*Z* = 1
*M**_r_* = 1203.04	*F*(000) = 627
Triclinic, *P*1	*D*_x_ = 1.331 Mg m^−^^3^
Hall symbol: -P 1	Mo *K*α radiation, λ = 0.71073 Å
*a* = 9.2832 (19) Å	Cell parameters from 4812 reflections
*b* = 12.744 (3) Å	θ = 4.6–22.4°
*c* = 13.317 (3) Å	µ = 0.37 mm^−^^1^
α = 84.55 (3)°	*T* = 295 K
β = 79.56 (3)°	Block, yellow
γ = 75.87 (3)°	0.28 × 0.27 × 0.26 mm
*V* = 1500.4 (6) Å^3^	

### Data collection

**Table:**

Bruker APEX CCD diffractometer	6832 independent reflections
Radiation source: fine-focus sealed tube	5066 reflections with *I* > 2σ(*I*)
graphite	*R*_int_ = 0.039
φ and ω scans	θ_max_ = 27.5°, θ_min_ = 3.1°
Absorption correction: multi-scan (*SADABS*; Sheldrick, 1996)	*h* = −12→11
*T*_min_ = 0.796, *T*_max_ = 0.808	*k* = −16→16
15862 measured reflections	*l* = −17→17

### Refinement

**Table:**

Refinement on *F*^2^	Primary atom site location: structure-invariant direct methods
Least-squares matrix: full	Secondary atom site location: difference Fourier map
*R*[*F*^2^ > 2σ(*F*^2^)] = 0.062	Hydrogen site location: inferred from neighbouring sites
*wR*(*F*^2^) = 0.188	H-atom parameters constrained
*S* = 0.84	*w* = 1/[σ^2^(*F*_o_^2^) + (0.1228*P*)^2^ + 1.5062*P*] where *P* = (*F*_o_^2^ + 2*F*_c_^2^)/3
6832 reflections	(Δ/σ)_max_ < 0.001
376 parameters	Δρ_max_ = 0.34 e Å^−^^3^
0 restraints	Δρ_min_ = −0.62 e Å^−^^3^

### Fractional atomic coordinates and isotropic or equivalent isotropic displacement parameters (Å^2^)

**Table:**

	*x*	*y*	*z*	*U*_iso_*/*U*_eq_	
Mn1	1.0000	0.5000	0.0000	0.02471 (16)	
N3	0.9796 (3)	0.35883 (19)	−0.06887 (19)	0.0422 (6)	
N1	1.1493 (3)	0.39905 (19)	0.09564 (18)	0.0412 (5)	
N5	0.8035 (3)	0.4910 (2)	0.11543 (19)	0.0455 (6)	
N2	1.2826 (3)	0.2585 (2)	0.1725 (2)	0.0477 (6)	
N4	0.9231 (3)	0.2058 (2)	−0.0898 (2)	0.0540 (7)	
N6	0.6644 (3)	0.4632 (2)	0.2627 (2)	0.0558 (7)	
C11	0.7996 (4)	0.4368 (3)	0.2039 (2)	0.0530 (8)	
H11A	0.8813	0.3859	0.2237	0.064*	
C1	1.1700 (3)	0.2942 (2)	0.1194 (2)	0.0429 (6)	
H1A	1.1128	0.2505	0.1014	0.051*	
C21	0.8943 (4)	0.2915 (2)	−0.0328 (2)	0.0470 (7)	
H21A	0.8219	0.3022	0.0258	0.056*	
C5	1.2858 (4)	0.1390 (3)	0.3294 (3)	0.0535 (8)	
C4	1.3311 (4)	0.1481 (3)	0.2159 (3)	0.0581 (9)	
H4A	1.4399	0.1249	0.1992	0.070*	
H4B	1.2880	0.0997	0.1849	0.070*	
C2	1.2564 (4)	0.4302 (3)	0.1362 (3)	0.0561 (8)	
H2A	1.2699	0.5005	0.1320	0.067*	
C25	0.7194 (5)	0.1279 (3)	−0.1260 (3)	0.0606 (9)	
C3	1.3391 (4)	0.3448 (3)	0.1830 (3)	0.0605 (9)	
H3A	1.4192	0.3448	0.2161	0.073*	
C22	1.0651 (4)	0.3146 (3)	−0.1548 (3)	0.0706 (11)	
H22A	1.1367	0.3447	−0.1981	0.085*	
C15	0.6994 (5)	0.4471 (3)	0.4434 (3)	0.0681 (11)	
C14	0.6202 (5)	0.4179 (4)	0.3652 (3)	0.0761 (12)	
H14A	0.6417	0.3396	0.3635	0.091*	
H14B	0.5124	0.4437	0.3856	0.091*	
C12	0.6625 (4)	0.5563 (3)	0.1181 (3)	0.0623 (9)	
H12A	0.6305	0.6048	0.0654	0.075*	
C23	1.0310 (5)	0.2205 (4)	−0.1686 (4)	0.0799 (14)	
H23A	1.0736	0.1748	−0.2219	0.096*	
C13	0.5767 (4)	0.5398 (4)	0.2083 (3)	0.0712 (11)	
H13A	0.4766	0.5743	0.2293	0.085*	
C17	0.7849 (6)	0.5760 (6)	0.5238 (5)	0.1018 (18)	
H17A	0.7962	0.6461	0.5272	0.122*	
C24	0.8506 (5)	0.1142 (3)	−0.0708 (3)	0.0695 (11)	
H24A	0.8163	0.1052	0.0020	0.083*	
H24B	0.9245	0.0487	−0.0919	0.083*	
C20	0.7495 (7)	0.3705 (5)	0.5150 (4)	0.1027 (17)	
H20A	0.7380	0.3004	0.5128	0.123*	
C27	0.6155 (7)	0.1387 (5)	−0.2792 (4)	0.0995 (17)	
H27A	0.6293	0.1409	−0.3502	0.119*	
C29	0.4569 (6)	0.1404 (5)	−0.1228 (6)	0.115 (2)	
H29A	0.3617	0.1422	−0.0851	0.138*	
C26	0.7371 (6)	0.1290 (4)	−0.2298 (4)	0.0850 (13)	
H26A	0.8328	0.1233	−0.2681	0.102*	
C10	1.1399 (5)	0.1396 (4)	0.3710 (4)	0.0858 (13)	
H10A	1.0682	0.1489	0.3284	0.103*	
C6	1.3894 (5)	0.1264 (4)	0.3928 (3)	0.0857 (14)	
H6A	1.4888	0.1274	0.3660	0.103*	
C18	0.8349 (7)	0.4956 (8)	0.5945 (5)	0.121 (2)	
H18A	0.8817	0.5106	0.6457	0.145*	
C28	0.4775 (7)	0.1447 (4)	−0.2246 (6)	0.0987 (17)	
H28A	0.3957	0.1520	−0.2581	0.118*	
C9	1.0982 (9)	0.1267 (5)	0.4738 (5)	0.124 (2)	
H9A	0.9982	0.1284	0.5013	0.149*	
C7	1.3462 (9)	0.1120 (6)	0.4965 (4)	0.134 (3)	
H7A	1.4173	0.1026	0.5397	0.161*	
C16	0.7176 (5)	0.5507 (4)	0.4475 (3)	0.0757 (12)	
H16A	0.6844	0.6040	0.3988	0.091*	
C30	0.5782 (6)	0.1331 (4)	−0.0734 (4)	0.0963 (16)	
H30A	0.5628	0.1318	−0.0024	0.116*	
C8	1.2031 (11)	0.1114 (5)	0.5363 (5)	0.139 (3)	
H8A	1.1756	0.1006	0.6065	0.167*	
C19	0.8155 (9)	0.3944 (8)	0.5892 (6)	0.132 (3)	
H19A	0.8484	0.3405	0.6376	0.159*	
Cl1	0.79576 (10)	0.12692 (7)	0.22679 (7)	0.0580 (2)	
O1	0.9416 (3)	0.1287 (3)	0.1713 (2)	0.0833 (9)	
O3	0.6900 (3)	0.2104 (3)	0.1833 (3)	0.0930 (10)	
O4	0.7860 (4)	0.1465 (3)	0.3315 (3)	0.1030 (11)	
O2	0.7630 (4)	0.0272 (3)	0.2180 (3)	0.1076 (12)	

### Atomic displacement parameters (Å^2^)

**Table:**

	*U*^11^	*U*^22^	*U*^33^	*U*^12^	*U*^13^	*U*^23^
Mn1	0.0268 (3)	0.0249 (3)	0.0220 (3)	−0.00701 (19)	−0.00161 (19)	−0.00076 (18)
N3	0.0437 (13)	0.0387 (13)	0.0442 (13)	−0.0072 (10)	−0.0073 (11)	−0.0077 (10)
N1	0.0440 (13)	0.0419 (13)	0.0386 (12)	−0.0113 (10)	−0.0092 (10)	0.0017 (10)
N5	0.0448 (14)	0.0474 (14)	0.0433 (14)	−0.0153 (11)	0.0038 (11)	−0.0063 (11)
N2	0.0529 (15)	0.0432 (14)	0.0461 (14)	−0.0068 (11)	−0.0154 (12)	0.0042 (11)
N4	0.0567 (16)	0.0424 (14)	0.0689 (18)	−0.0108 (12)	−0.0206 (14)	−0.0142 (13)
N6	0.0570 (17)	0.0605 (17)	0.0476 (15)	−0.0247 (14)	0.0190 (13)	−0.0155 (13)
C11	0.0571 (19)	0.0523 (18)	0.0439 (17)	−0.0162 (15)	0.0123 (15)	−0.0041 (14)
C1	0.0465 (16)	0.0396 (15)	0.0440 (16)	−0.0131 (12)	−0.0094 (13)	0.0019 (12)
C21	0.0607 (19)	0.0396 (15)	0.0444 (16)	−0.0158 (14)	−0.0116 (14)	−0.0047 (13)
C5	0.061 (2)	0.0446 (17)	0.0525 (19)	−0.0051 (15)	−0.0179 (16)	0.0088 (14)
C4	0.073 (2)	0.0418 (17)	0.0540 (19)	−0.0006 (16)	−0.0169 (17)	0.0063 (14)
C2	0.068 (2)	0.0441 (17)	0.064 (2)	−0.0168 (15)	−0.0312 (18)	0.0044 (15)
C25	0.072 (2)	0.0407 (17)	0.078 (3)	−0.0212 (16)	−0.020 (2)	−0.0092 (17)
C3	0.067 (2)	0.0539 (19)	0.070 (2)	−0.0156 (17)	−0.0364 (19)	0.0025 (17)
C22	0.058 (2)	0.085 (3)	0.074 (2)	−0.030 (2)	0.0125 (19)	−0.042 (2)
C15	0.070 (2)	0.072 (2)	0.051 (2)	−0.017 (2)	0.0224 (18)	−0.0111 (18)
C14	0.090 (3)	0.083 (3)	0.053 (2)	−0.042 (2)	0.031 (2)	−0.015 (2)
C12	0.0468 (19)	0.075 (2)	0.059 (2)	−0.0089 (17)	−0.0002 (16)	−0.0042 (18)
C23	0.061 (2)	0.091 (3)	0.094 (3)	−0.027 (2)	0.013 (2)	−0.062 (3)
C13	0.0459 (19)	0.088 (3)	0.071 (3)	−0.0093 (19)	0.0129 (18)	−0.022 (2)
C17	0.083 (3)	0.130 (5)	0.096 (4)	−0.038 (3)	0.016 (3)	−0.046 (4)
C24	0.090 (3)	0.0420 (18)	0.089 (3)	−0.0231 (18)	−0.038 (2)	−0.0039 (18)
C20	0.113 (4)	0.096 (4)	0.080 (3)	−0.004 (3)	0.000 (3)	0.007 (3)
C27	0.128 (5)	0.104 (4)	0.093 (4)	−0.057 (4)	−0.052 (3)	0.007 (3)
C29	0.064 (3)	0.133 (5)	0.147 (6)	−0.014 (3)	−0.009 (4)	−0.039 (4)
C26	0.085 (3)	0.103 (4)	0.085 (3)	−0.049 (3)	−0.026 (3)	0.006 (3)
C10	0.072 (3)	0.092 (3)	0.092 (3)	−0.025 (2)	−0.005 (2)	0.004 (3)
C6	0.074 (3)	0.105 (4)	0.067 (3)	0.010 (2)	−0.030 (2)	0.000 (2)
C18	0.073 (3)	0.204 (8)	0.075 (4)	−0.011 (5)	−0.002 (3)	−0.034 (5)
C28	0.081 (3)	0.076 (3)	0.151 (6)	−0.018 (3)	−0.052 (4)	−0.009 (3)
C9	0.129 (5)	0.105 (4)	0.109 (5)	−0.022 (4)	0.041 (4)	0.014 (4)
C7	0.149 (6)	0.156 (6)	0.066 (3)	0.046 (5)	−0.051 (4)	−0.006 (3)
C16	0.085 (3)	0.076 (3)	0.064 (2)	−0.027 (2)	0.010 (2)	−0.016 (2)
C30	0.086 (3)	0.105 (4)	0.098 (4)	−0.018 (3)	−0.006 (3)	−0.038 (3)
C8	0.188 (8)	0.111 (5)	0.061 (3)	0.034 (5)	0.017 (4)	0.028 (3)
C19	0.126 (6)	0.150 (7)	0.096 (5)	0.005 (5)	−0.015 (4)	0.009 (5)
Cl1	0.0584 (5)	0.0493 (5)	0.0669 (5)	−0.0173 (4)	−0.0080 (4)	0.0016 (4)
O1	0.0582 (16)	0.100 (2)	0.091 (2)	−0.0260 (16)	−0.0023 (15)	0.0013 (18)
O3	0.0729 (19)	0.084 (2)	0.107 (2)	0.0009 (16)	−0.0150 (18)	0.0226 (18)
O4	0.105 (3)	0.133 (3)	0.073 (2)	−0.028 (2)	−0.0112 (19)	−0.020 (2)
O2	0.127 (3)	0.0634 (19)	0.142 (3)	−0.047 (2)	−0.009 (3)	−0.010 (2)

### Geometric parameters (Å, °)

**Table:**

Mn1—N1	2.158 (2)	C14—H14B	0.9700
Mn1—N3	2.158 (2)	C12—C13	1.346 (5)
Mn1—N5	2.181 (2)	C12—H12A	0.9300
N3—C21	1.307 (4)	C23—H23A	0.9300
N3—C22	1.353 (4)	C13—H13A	0.9300
N1—C1	1.319 (4)	C17—C18	1.376 (9)
N1—C2	1.366 (4)	C17—C16	1.384 (7)
N5—C11	1.307 (4)	C17—H17A	0.9300
N5—C12	1.366 (4)	C24—H24A	0.9700
N2—C1	1.332 (4)	C24—H24B	0.9700
N2—C3	1.358 (4)	C20—C19	1.346 (10)
N2—C4	1.465 (4)	C20—H20A	0.9300
N4—C21	1.336 (4)	C27—C28	1.342 (8)
N4—C23	1.346 (5)	C27—C26	1.382 (7)
N4—C24	1.464 (4)	C27—H27A	0.9300
N6—C11	1.338 (4)	C29—C28	1.333 (9)
N6—C13	1.353 (5)	C29—C30	1.384 (8)
N6—C14	1.458 (4)	C29—H29A	0.9300
C11—H11A	0.9300	C26—H26A	0.9300
C1—H1A	0.9300	C10—C9	1.359 (8)
C21—H21A	0.9300	C10—H10A	0.9300
C5—C6	1.362 (5)	C6—C7	1.375 (7)
C5—C10	1.366 (6)	C6—H6A	0.9300
C5—C4	1.495 (5)	C18—C19	1.355 (10)
C4—H4A	0.9700	C18—H18A	0.9300
C4—H4B	0.9700	C28—H28A	0.9300
C2—C3	1.341 (5)	C9—C8	1.361 (11)
C2—H2A	0.9300	C9—H9A	0.9300
C25—C30	1.358 (6)	C7—C8	1.339 (10)
C25—C26	1.361 (6)	C7—H7A	0.9300
C25—C24	1.501 (5)	C16—H16A	0.9300
C3—H3A	0.9300	C30—H30A	0.9300
C22—C23	1.349 (5)	C8—H8A	0.9300
C22—H22A	0.9300	C19—H19A	0.9300
C15—C20	1.356 (7)	Cl1—O2	1.397 (3)
C15—C16	1.379 (6)	Cl1—O3	1.418 (3)
C15—C14	1.500 (6)	Cl1—O4	1.422 (3)
C14—H14A	0.9700	Cl1—O1	1.424 (3)
			
N1—Mn1—N1^i^	180.000 (1)	N6—C14—H14B	109.0
N1—Mn1—N3^i^	89.45 (9)	C15—C14—H14B	109.0
N1^i^—Mn1—N3^i^	90.55 (9)	H14A—C14—H14B	107.8
N1—Mn1—N3	90.55 (9)	C13—C12—N5	109.7 (4)
N1^i^—Mn1—N3	89.45 (9)	C13—C12—H12A	125.2
N3^i^—Mn1—N3	180.00 (12)	N5—C12—H12A	125.2
N1—Mn1—N5	91.52 (10)	N4—C23—C22	106.5 (3)
N1^i^—Mn1—N5	88.48 (10)	N4—C23—H23A	126.8
N3^i^—Mn1—N5	89.34 (10)	C22—C23—H23A	126.8
N3—Mn1—N5	90.66 (10)	C12—C13—N6	106.7 (3)
N1—Mn1—N5^i^	88.48 (10)	C12—C13—H13A	126.6
N1^i^—Mn1—N5^i^	91.52 (10)	N6—C13—H13A	126.6
N3^i^—Mn1—N5^i^	90.66 (10)	C18—C17—C16	118.8 (6)
N3—Mn1—N5^i^	89.34 (10)	C18—C17—H17A	120.6
N5—Mn1—N5^i^	180.00 (14)	C16—C17—H17A	120.6
C21—N3—C22	104.8 (3)	N4—C24—C25	113.3 (3)
C21—N3—Mn1	128.3 (2)	N4—C24—H24A	108.9
C22—N3—Mn1	126.6 (2)	C25—C24—H24A	108.9
C1—N1—C2	104.6 (3)	N4—C24—H24B	108.9
C1—N1—Mn1	129.2 (2)	C25—C24—H24B	108.9
C2—N1—Mn1	125.8 (2)	H24A—C24—H24B	107.7
C11—N5—C12	105.0 (3)	C19—C20—C15	121.2 (7)
C11—N5—Mn1	127.9 (2)	C19—C20—H20A	119.4
C12—N5—Mn1	126.2 (2)	C15—C20—H20A	119.4
C1—N2—C3	107.1 (3)	C28—C27—C26	119.9 (5)
C1—N2—C4	126.4 (3)	C28—C27—H27A	120.0
C3—N2—C4	126.4 (3)	C26—C27—H27A	120.0
C21—N4—C23	106.5 (3)	C28—C29—C30	119.5 (6)
C21—N4—C24	126.7 (3)	C28—C29—H29A	120.3
C23—N4—C24	126.8 (3)	C30—C29—H29A	120.3
C11—N6—C13	106.5 (3)	C25—C26—C27	121.1 (5)
C11—N6—C14	126.6 (4)	C25—C26—H26A	119.4
C13—N6—C14	126.8 (3)	C27—C26—H26A	119.4
N5—C11—N6	112.1 (3)	C9—C10—C5	120.9 (6)
N5—C11—H11A	124.0	C9—C10—H10A	119.5
N6—C11—H11A	124.0	C5—C10—H10A	119.5
N1—C1—N2	111.8 (3)	C5—C6—C7	119.7 (5)
N1—C1—H1A	124.1	C5—C6—H6A	120.2
N2—C1—H1A	124.1	C7—C6—H6A	120.2
N3—C21—N4	112.2 (3)	C19—C18—C17	119.8 (7)
N3—C21—H21A	123.9	C19—C18—H18A	120.1
N4—C21—H21A	123.9	C17—C18—H18A	120.1
C6—C5—C10	118.9 (4)	C29—C28—C27	120.5 (5)
C6—C5—C4	120.9 (4)	C29—C28—H28A	119.8
C10—C5—C4	120.2 (4)	C27—C28—H28A	119.8
N2—C4—C5	112.9 (3)	C10—C9—C8	119.8 (6)
N2—C4—H4A	109.0	C10—C9—H9A	120.1
C5—C4—H4A	109.0	C8—C9—H9A	120.1
N2—C4—H4B	109.0	C8—C7—C6	120.8 (6)
C5—C4—H4B	109.0	C8—C7—H7A	119.6
H4A—C4—H4B	107.8	C6—C7—H7A	119.6
C3—C2—N1	110.3 (3)	C15—C16—C17	120.5 (5)
C3—C2—H2A	124.9	C15—C16—H16A	119.8
N1—C2—H2A	124.9	C17—C16—H16A	119.8
C30—C25—C26	117.2 (4)	C25—C30—C29	121.8 (5)
C30—C25—C24	120.8 (4)	C25—C30—H30A	119.1
C26—C25—C24	121.9 (4)	C29—C30—H30A	119.1
C2—C3—N2	106.3 (3)	C7—C8—C9	119.9 (5)
C2—C3—H3A	126.9	C7—C8—H8A	120.1
N2—C3—H3A	126.9	C9—C8—H8A	120.1
C23—C22—N3	110.0 (3)	C20—C19—C18	121.1 (7)
C23—C22—H22A	125.0	C20—C19—H19A	119.5
N3—C22—H22A	125.0	C18—C19—H19A	119.5
C20—C15—C16	118.7 (5)	O2—Cl1—O3	109.1 (2)
C20—C15—C14	119.2 (5)	O2—Cl1—O4	110.3 (2)
C16—C15—C14	122.1 (4)	O3—Cl1—O4	108.7 (2)
N6—C14—C15	113.1 (3)	O2—Cl1—O1	109.8 (2)
N6—C14—H14A	109.0	O3—Cl1—O1	107.8 (2)
C15—C14—H14A	109.0	O4—Cl1—O1	111.0 (2)
			
N1—Mn1—N3—C21	80.4 (3)	C4—N2—C3—C2	−176.4 (3)
N1^i^—Mn1—N3—C21	−99.6 (3)	C21—N3—C22—C23	−0.7 (5)
N5—Mn1—N3—C21	−11.1 (3)	Mn1—N3—C22—C23	173.3 (3)
N5^i^—Mn1—N3—C21	168.9 (3)	C11—N6—C14—C15	67.0 (5)
N1—Mn1—N3—C22	−92.2 (3)	C13—N6—C14—C15	−113.2 (5)
N1^i^—Mn1—N3—C22	87.8 (3)	C20—C15—C14—N6	−138.7 (4)
N5—Mn1—N3—C22	176.3 (3)	C16—C15—C14—N6	44.1 (5)
N5^i^—Mn1—N3—C22	−3.7 (3)	C11—N5—C12—C13	0.0 (4)
N3^i^—Mn1—N1—C1	161.6 (3)	Mn1—N5—C12—C13	−169.7 (3)
N3—Mn1—N1—C1	−18.4 (3)	C21—N4—C23—C22	1.0 (5)
N5—Mn1—N1—C1	72.3 (3)	C24—N4—C23—C22	−179.5 (4)
N5^i^—Mn1—N1—C1	−107.7 (3)	N3—C22—C23—N4	−0.2 (5)
N3^i^—Mn1—N1—C2	−26.1 (3)	N5—C12—C13—N6	−0.3 (5)
N3—Mn1—N1—C2	153.9 (3)	C11—N6—C13—C12	0.4 (4)
N5—Mn1—N1—C2	−115.4 (3)	C14—N6—C13—C12	−179.4 (4)
N5^i^—Mn1—N1—C2	64.6 (3)	C21—N4—C24—C25	93.9 (4)
N1—Mn1—N5—C11	−2.1 (3)	C23—N4—C24—C25	−85.5 (5)
N1^i^—Mn1—N5—C11	177.9 (3)	C30—C25—C24—N4	−117.2 (4)
N3^i^—Mn1—N5—C11	−91.6 (3)	C26—C25—C24—N4	66.7 (5)
N3—Mn1—N5—C11	88.4 (3)	C16—C15—C20—C19	0.2 (8)
N1—Mn1—N5—C12	165.2 (3)	C14—C15—C20—C19	−177.2 (5)
N1^i^—Mn1—N5—C12	−14.8 (3)	C30—C25—C26—C27	2.0 (7)
N3^i^—Mn1—N5—C12	75.7 (3)	C24—C25—C26—C27	178.2 (4)
N3—Mn1—N5—C12	−104.3 (3)	C28—C27—C26—C25	−1.4 (8)
C12—N5—C11—N6	0.3 (4)	C6—C5—C10—C9	−0.7 (7)
Mn1—N5—C11—N6	169.7 (2)	C4—C5—C10—C9	177.2 (5)
C13—N6—C11—N5	−0.5 (4)	C10—C5—C6—C7	1.5 (7)
C14—N6—C11—N5	179.3 (3)	C4—C5—C6—C7	−176.3 (5)
C2—N1—C1—N2	0.2 (4)	C16—C17—C18—C19	−0.9 (8)
Mn1—N1—C1—N2	173.80 (19)	C30—C29—C28—C27	2.1 (10)
C3—N2—C1—N1	−0.5 (4)	C26—C27—C28—C29	−0.7 (9)
C4—N2—C1—N1	176.5 (3)	C5—C10—C9—C8	−1.0 (9)
C22—N3—C21—N4	1.4 (4)	C5—C6—C7—C8	−0.7 (10)
Mn1—N3—C21—N4	−172.5 (2)	C20—C15—C16—C17	−0.4 (6)
C23—N4—C21—N3	−1.6 (4)	C14—C15—C16—C17	176.9 (4)
C24—N4—C21—N3	178.9 (3)	C18—C17—C16—C15	0.8 (7)
C1—N2—C4—C5	−105.6 (4)	C26—C25—C30—C29	−0.6 (8)
C3—N2—C4—C5	70.8 (5)	C24—C25—C30—C29	−176.8 (5)
C6—C5—C4—N2	−106.6 (4)	C28—C29—C30—C25	−1.5 (9)
C10—C5—C4—N2	75.7 (5)	C6—C7—C8—C9	−1.0 (11)
C1—N1—C2—C3	0.2 (4)	C10—C9—C8—C7	1.8 (11)
Mn1—N1—C2—C3	−173.7 (2)	C15—C20—C19—C18	−0.3 (10)
N1—C2—C3—N2	−0.5 (4)	C17—C18—C19—C20	0.7 (10)
C1—N2—C3—C2	0.6 (4)		

Symmetry codes: (i) −*x*+2, −*y*+1, −*z*.

### Hydrogen-bond geometry (Å, °)

**Table:**

*D*—H···*A*	*D*—H	H···*A*	*D*···*A*	*D*—H···*A*
C1—H1A···O1	0.93	2.49	3.286 (4)	144
C14—H14A···O4	0.97	2.53	3.461 (6)	160
C21—H21A···O3	0.93	2.56	3.371 (5)	145
C24—H24B···O1^ii^	0.97	2.53	3.469 (5)	164

Symmetry codes:  (ii) −*x*+2, −*y*, −*z*.
